# What Have We Learned from Molecularly Informed Clinical Trials on Thymomas and Thymic Carcinomas—Current Status and Future Directions?

**DOI:** 10.3390/cancers16020416

**Published:** 2024-01-18

**Authors:** Rohan Maniar, Patrick J. Loehrer

**Affiliations:** Division of Hematology & Oncology, Indiana University School of Medicine, Indianapolis, IN 46202, USA; rmaniar@iu.edu

**Keywords:** thymoma, thymic carcinoma, targeted therapies

## Abstract

**Simple Summary:**

Thymoma and thymic carcinoma are uncommon tumors collectively known as thymic epithelial tumors (TETs) that arise from the thymus gland and are characterized by a unique pattern of spread. For patients with early-stage disease, surgery remains the primary treatment, while systemic chemotherapy is typically employed for more advanced and metastatic disease. While targeted therapies have revolutionized the treatment of many cancers, only a handful of these agents have shown efficacy against TETs. In this review, we describe the landscape of molecular alterations and targeted therapies that were previously studied and describe some of the ongoing studies that have the potential to advance our understanding and improve outcomes for patients with TETs.

**Abstract:**

Thymic epithelial tumors (TETs), which include thymomas and thymic carcinomas, are a rare, heterogeneous group of malignancies that originate from the thymus gland. As an important organ of immune cell development, thymic tumors, particularly thymomas, are often associated with paraneoplastic autoimmune disorders. The advances in targeted therapies for both solid and hematologic malignancies have resulted in improved patient outcomes, including better and more durable efficacy and improved toxicity. Targeted therapies have also been investigated in the treatment of TETs, though the results have largely been modest. These have included somatostatin-receptor-targeting therapies, KIT- and EGFR-directed tyrosine kinase inhibitors, epigenetic modulators, anti-angiogenesis agents, and agents targeting the cell proliferation and survival pathways and cell cycle regulators. Numerous investigated treatments have failed or underperformed due to a lack of a strong biomarker of efficacy. Ongoing trials are attempting to expand on previous experiences, including the exploration of effective drugs in early-stage disease. Novel combination therapy strategies are also undergoing evaluation, with the goal of augmenting efficacy and understanding the toxicity while expanding the biomarkers of efficacy and safety. With advances in technology to improve target identification and drug delivery, old targets may become new opportunities, and the subsequently developed drugs may find their place in the treatment of thymic tumors.

## 1. Introduction

The introduction of imatinib, which is an orally administered tyrosine kinase inhibitor (TKI) that targets the BCR-ABL fusion in Philadelphia chromosome-positive chronic myeloid leukemia in 2001, was revolutionary, as highlighted by a cover piece in Time Magazine as a new weapon in the war against cancer [1,2]. Prior to that, the anti-HER2 monoclonal antibody, namely, trastuzumab, received FDA approval in 1998 for the treatment of HER2-positive breast cancer [3]. And so began the era of precision oncology, driven by our growing understanding of molecular alterations and foundational advances in target identification and drug development technologies. Since that time, a growing arsenal of targeted therapies has established their place in the treatment of cancers.

**Figure 1:**
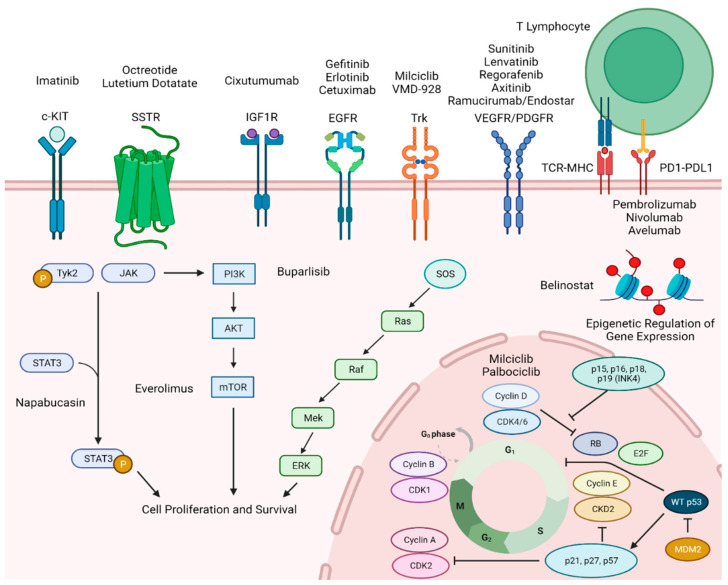
Molecular pathways involved in TET tumorigenesis and relevant targeted and immune-based therapies previously studied or currently undergoing clinical evaluation. SSTR—somatostatin receptor, IGF1R—insulin-like growth factor 1 receptor, EGFR—epidermal growth factor receptor, VEGFR—vascular endothelial growth factor receptor, PDGFR—platelet derived growth factor receptor, PD-1/PD-L1—programmed death/programmed death ligand-1, TCR—T cell receptor, MHC—major histocompatibility complex.

Thymic epithelial tumors (TETs), including thymomas and thymic carcinomas, are a rare, heterogeneous group of malignancies that arise from the thymus gland, which serves a crucial role in T-cell maturation, particularly in early life. Paraneoplastic autoimmune disorders, such as myasthenia gravis and pure red cell aplasia, are commonly associated with thymomas, which are likely the result of dysfunctional T-cell development [4,5]. Thymic tumors are the most common malignancy of the anterior mediastinum, with a propensity for metastatic spread to the pleura and pericardium [5]. Despite these similarities, thymomas and thymic carcinomas represent biologically distinct entities with unique clinical characteristics and a disparate natural history. Knowledge of the genetic and molecular framework underlying the etiology of TETs has long been limited, in part owing to the rarity of these diseases and lack of preclinical models. Despite these obstacles, many studies attempted to interrogate thymic tumors in a genomic, transcriptomic, and proteomic fashion to uncover potential targetable alterations (summarized in Figure 1). Prior comprehensive analyses revealed distinct molecular alterations, including GTF2I, TP53/cell cycle regulators, PTEN/PI3K/mTOR, and RAS pathways while generally carrying a low tumor mutational burden [6,7,8,9]. Numerous clinical studies attempted to replicate the “magic bullet” of imatinib; however, no such agent has been found (summarized in Table 1). But times continue to change, technologies continue to evolve, and thus, hope remains that targeted therapies will find a meaningful place in the treatment of thymic tumors and provide tangible benefits for patients while limiting worrisome drug toxicities.

## 2. Somatostatin Receptor Targeting Therapies

Somatostatin receptor (SSTR) expression has been reported in well-differentiated neuroendocrine tumors of the GI tract, thyroid carcinoma, and pulmonary carcinoids [36]. Several studies have reported SSTR expression in thymic tumors [37,38]. Octreotide, which is an SST analog with high affinity to the SST_2_ subtype receptor, was evaluated in combination with prednisone in a case report relaying a complete remission and resolution of an associated paraneoplastic autoimmune disorder, namely, pure red cell aplasia, in a patient with thymoma [39]. Unsure whether the lymphodepleting effect of the prednisone diminished the tumor size, the Eastern Cooperative Oncology Group initiated a phase 2 trial that evaluated octreotide alone and in combination with prednisone in patients with recurrent or metastatic thymoma and thymic carcinoma that demonstrated activity on a radionuclide octreotide scan with measurable disease and confirmed single-agent activity [11].

Patients received short-acting octreotide (0.5 mg subcutaneously three times a day) for two cycles (1-month cycle), followed by interval imaging. If a patient experienced a complete or partial response (CR/PR), they continued the current treatment regimen. If a patient experienced stable disease (SD), they remained in the study with the addition of prednisone. Of the 38 evaluable patients (32 with thymoma, 5 with thymic carcinoma, 1 with thymic carcinoid), the overall response rate (ORR) was 31.6%, with 2 CRs and 10 PRs, and a disease control rate (DCR) of 68.4%. In total, 4 patients of the 38 achieved a PR with octreotide alone, and 2 and 4 patients experienced CRs and PRs, respectively, with the addition of prednisone. No patients with thymic carcinoma or thymic carcinoid experienced an objective response to therapy. The median progression-free survival (PFS) was 2 months for octreotide alone and 9.2 months for octreotide plus prednisone. As expected, differences in PFS were seen between thymoma and thymic carcinoma, with a median PFS of 8.8 and 4.5 months, respectively. Common grade 3 and 4 toxicities included diarrhea, hematologic toxicity, hyperglycemia, electrolyte disturbances, transaminitis, and acidosis. One patient who received octreotide and prednisone experienced a fatal infection without neutropenia. Overall, the study showed minimal activity with octreotide alone, which was generally augmented with the addition of prednisone to the treatment regimen. Long-acting octreotide was not available at the start of the trial, though subsequent studies and case reports have utilized this formulation for improved dose schedules.

A study of induction long-acting octreotide combined with prednisone explored their potential use in locally advanced or recurrent, unresectable TET patients with the goal of evaluating treatment responses and feasibility of surgery [40]. A total of 17 patients (15 with thymoma, 2 with thymic carcinoma) received intramuscular long-acting octreotide (30 mg every two weeks) with prednisone (0.6 mg/kg/day) for a maximum of 24 weeks, with an evaluation of disease status at 12 weeks. The study reported an ORR of 88%, with a median tumor volume reduction of 51% at week 12. Surgical resection was performed in 29% and 23% of patients after 12 and 24 weeks, respectively. The study reported no changes in pre-existing myasthenic symptoms compared with baseline. Grade 3 adverse events occurred in 17.6% of patients and included infection and pneumonia. One patient experienced pulmonary sepsis that was fatal and attributed to thymoma-associated immunodeficiency while on concurrent steroids.

Lutetium Lu 177 dotatate (Lutathera) is a peptide receptor radioligand therapy (PRRT) that showed efficacy in the treatment of gastroenteropancreatic neuroendocrine tumors expressing SSTRs [41]. Long-acting SSTR analogs and PRRT remain enticing therapeutic options that have yet to be meaningfully explored in select TET patients, though case reports and retrospective series offer glimpses of potential utility [42,43,44,45]. The LEVEL trial (NCT05918302) is an ongoing study of 177-Lu-edotreitude combined with everolimus, which is a mTOR inhibitor, versus everolimus alone for patients with SSTR expressing thymic neuroendocrine tumors [12]. Similarly, a phase 1 study (NCT04375267) is evaluating 177-Lu-DOTA-TATE in combination with the PARP inhibitor olaparib in SSTR-expressing thoracic tumors, with the goal of sensitizing the effects of ionizing radiation by targeting the DNA repair mechanisms of dependent tumor cells [13].

## 3. EGFR and c-KIT

Epidermal growth factor receptor (EGFR) is a member of the ErbB family of tyrosine kinase receptors and regulates epithelial cell function and homeostasis [46]. Aberrant EGFR signaling was identified in several human cancers, most commonly seen in glioblastoma multiforme (GBM) and non-small cell lung cancer (NSCLC) [46,47]. Alterations may include gene amplification or protein overexpression, as seen in GBM, or as mutations or in-frame deletions, which are more commonly seen in NSCLC. Increased EGFR expression was noted to confer poorer outcomes in head and neck, esophageal, and triple-negative breast cancer [48,49,50]. Numerous studies reported the overexpression of EGFR in thymomas via immunohistochemistry (IHC) analysis resulting in the clinical trial exploration of EGFR targeting agents [51,52,53,54].

Gefitinib and erlotinib are first-generation, small-molecule EGFR TKIs that work by reversibly binding to the kinase domain of the receptor [55]. Kurup et al. evaluated gefitinib in an unselected, heavily pre-treated patient population with metastatic thymic tumors. A total of 26 patients (19 with thymoma, 7 with thymic carcinoma) received gefitinib orally at 250 mg daily and reported no CRs, 1 PR and 14 patients with SD, resulting in an ORR of 4% and a DCR of 58% [14]. Serious adverse events included dyspnea, fatigue, anemia/thrombocytopenia, and myocardial infarction. Of note, the patient who achieved a PR experienced grade 2 pneumonitis after three cycles of treatment that subsequently resolved with steroids and discontinuation of their TKI. Genomic sequencing on five patients, including the patient who experienced a PR, did not reveal EGFR mutations.

Bendano et al. reported the results of a phase 2 study that evaluated erlotinib, which is an EGFR TKI, and bevacizumab, which is an anti-angiogenesis agent, in patients with recurrent thymoma and thymic carcinoma [15]. Similar to the gefitinib trial, an unselected and pretreated population was enrolled in this study. A total of 18 patients (11 with thymoma, 7 with thymic carcinoma) received erlotinib 150 mg daily with an anti-angiogenesis agent, bevacizumab 15 mg/kg intravenously on day 1 and every 21 days. This study reported no CRs or PRs, while 60% of patients experienced SD and 40% of patients experienced progressive disease. The treatments were generally well tolerated, with the most common grade 3 toxicities being acneiform rash, dyspnea, fatigue, pericardial tamponade, and aortic insufficiency. No grade 4 toxicities were seen in this study.

The results of these studies were largely disappointing, particularly as the clinical utility of these agents in NSCLC harboring EGFR mutations gained traction in the subsequent years. For thymic tumors, EGFR protein overexpression did not correlate with histologic subtype, and discrepancies between protein expression and gene amplification were noted [56]. Further studies to clarify these findings ultimately reported frequent protein overexpression without associated mutations. Case reports noted the potential use of cetuximab, which is an EGFR-targeting monoclonal antibody, in EGFR-expressing thymomas [57,58]. An ongoing phase 2 study (NCT01025089) is evaluating cetuximab in combination with platinum-based chemotherapy in patients with advanced thymic tumors, with the intent of surgical resection after neoadjuvant therapy [16].

*c-KIT* is a proto-oncogene that plays a critical role in cell proliferation, differentiation, and survival encoding for the receptor tyrosine kinase protein KIT (CD117) or mast/stem cell growth factor receptor (SCFR) [59]. These receptors are expressed on numerous cell types, including hematopoietic stem cells. In particular, the capacity to self-renew in cancer cells has been associated with mutations in *c-KIT*, resulting in constitutive activation [59]. Activating mutations were seen in a variety of cancers, including gastrointestinal stromal tumors (GIST), testicular seminomas, and extracutaneous melanomas [60,61,62]. The previously mentioned drug imatinib, having garnered success in the world of hematologic malignancies, demonstrated significant activity in the treatment of unresectable and metastatic GISTs with *c-KIT* mutations [63].

Several subsequent studies evaluated the IHC expression of c-KIT in thymic neoplasms, revealing positivity in 4% of thymomas and 80–86% of thymic carcinoma cases [64,65,66,67]. A case report of a thymic carcinoma patient who harbored an activating KIT mutation noted a clinical and radiographic response to imatinib therapy [68]. Though the response was short-lived, it served as the foundation for two small phase 2 studies in an unselected TET patient population. Salter et al. conducted a study of imatinib in 11 patients with advanced, unresectable thymic carcinoma showing no responses despite KIT overexpression in nine cases, as measured using IHC [17]. Tumor samples, however, were not analyzed for activating mutations in this study. Giaccone et al. evaluated imatinib in seven TET patients (2 with World Health Organization [WHO] B3 thymomas, 5 with thymic carcinomas) with advanced, unresectable disease, noting no responses in any of the patients and stable disease as the best response among the thymoma patients [18]. KIT IHC and mutational analysis of the tumors noted positivity in only one of the thymoma samples, while the remainder were either negative or testing was not available. Subsequent studies noted the discordance of KIT overexpression with the presence of activating mutations [69]. Mutations in *c-KIT* were generally seen in approximately 9% of thymic carcinoma cases based on the The Cancer Genome Atlas Program analysis [6].

Despite the best efforts to build preclinical and translational models to support the use of EGFR and KIT TKIs, these drugs ultimately showed limited activity across the spectrum of thymic tumors. Later studies would go on to clarify the need for sensitizing mutations to see clinical efficacy with these agents. While disappointing, the seemingly ubiquitous presence of EGFR and KIT amplification should serve as a starting point for building novel agents that have specificity for thymic tumor cells, including the potential use of antibody-drug conjugates, bispecific antibodies, and adoptive cell therapies.

## 4. Epigenetic Alterations and Associated Therapies

Epigenetics is a field of study that was originally defined by C.H. Waddington in 1942 as “the casual interactions between genes and their products, which bring the phenotype into being” [70]. Research in the field of epigenetics, namely, the regulation and organization of the genomic structure and its impact on gene expression, has continued to grow, as highlighted by the fundamental understanding that these mechanisms are essential for normal tissue development and appropriate tissue-specific gene expression. We have also gained greater insight into the role of epigenetics in promoting tumorigenesis, including the overexpression of oncogenes, inactivation of suppressor genes, and modulation of the tumor microenvironment (TME). Alterations in the epigenetic pathways governing cellular machinery can include DNA methylation, histone modification, nucleosome remodeling, and RNA interference via microRNA (miRNA) and long non-coding RNA [71].

Numerous epigenetic abnormalities have been reported across all histologic subtypes of TETs [72]. Wang et al. reported on molecular profiling using target-capture sequencing of 78 advanced TET patients (31 with thymoma, 47 with thymic carcinoma), including paired tissue and blood samples, and noted mutations in histone modification and chromatin remodeling genes (*BAP1*, *ASXL1*, *SETD2*, *SMARCA4*), and DNA methylation genes (*TET2*, *DNMT3A*, *WT1*) [73]. Mutations in these pathways were more commonly seen in thymic carcinoma compared with thymoma (38% vs. 10%), though the presence of mutations did not appear to confer worse outcomes.

Histone deacetylases (HDACs) regulate the acetylation of histone and non-histone proteins, resulting in structural chromatin changes that affect the transcriptional activity [74]. Belinostat, which is a pan-HDAC inhibitor, received FDA approval in 2014 for the treatment of relapsed and refractory peripheral T-cell lymphoma. While not the first HDAC inhibitor approved in this therapeutic area, it showed promise with a very manageable toxicity profile [74]. Belinostat was initially evaluated in a study of 41 TET patients (25 with thymoma, 16 with thymic carcinoma) with advanced disease who had previously been treated with systemic therapy [19]. The response rates were 8% and 0% in thymoma and thymic carcinoma patients, respectively, with disease control seen in 79% of thymoma patients and 50% of thymic carcinoma patients. The median PFSs were 11.4 and 2.7 months in thymoma and thymic carcinoma, respectively. Though somewhat disappointing in its efficacy as a single agent, the drug demonstrated a manageable safety profile, with the most common adverse events of nausea, vomiting, and fatigue. Three patients required discontinuation due to complications with the drug, including polymyositis, sepsis, and QTc prolongation. Correlative analysis was conducted to identify biomarkers of response, profile the impact of HDAC inhibition of the peripheral immune system, and identify the impact of inhibition of angiogenesis through repression of hypoxia-induced angiogenic factors. Peripheral blood mononuclear cells (PBMCs) were evaluated via flow cytometry for protein and tubulin-specific hyperacetylation. While global protein hyperacetylation was seen in all patients in the study, the degree of acetylation did not appear to correlate with the response, survival, or toxicity. Regarding immune profiling, the majority of patients in the study saw an expansion of peripheral HLA-DR^+^ T regulatory cells (Tregs) during treatment, which did not correlate with the response. Similarly, belinostat had a significant impact on circulating angiogenic factors, placental-derived growth factor, and basic fibroblast growth factor, though no clinical correlation was noted.

A phase I/II study evaluated the addition of belinostat to systemic therapy with cisplatin, doxorubicin, and cyclophosphamide (PAC) based on preclinical data, which suggested increased chemosensitivity in the setting of altered gene expression and targeting of chemo-resistant tumor clones harboring an altered chromatin state [20,75]. A total of 26 TET patients (12 with thymoma, 14 with thymic carcinoma) who were treatment naïve in the advanced setting received belinostat in combination with PAC. The study reported an ORR of 64% in thymoma and 21% in thymic carcinoma. The safety profile of combination therapy was generally similar to previous studies of these agents. As demonstrated in the previous study of belinostat as a single agent, significant increases in hyperacetylation of PBMCs did not correlate with the response or survival. The expression of p21 in CD14^+^ cells did appear to increase after exposure to belinostat, both alone and in the combination treatment, and was associated with a trend toward improved PFS and OS. The study also noted a decline in circulating Tregs that was associated with belinostat exposure, both alone and in combination with PAC. The breadth of decline for Tregs was significantly larger in patients who responded compared with those who did not respond to treatment, and those patients who experienced greater Treg decline after initiation of combination therapy had an improved PFS compared with those who did not achieve the same effect. Patients with a higher frequency of CTLA4^+^ Tregs at baseline were noted to have worse overall survival (OS) compared with those with lower frequency in the peripheral blood. Surprisingly, while exposure to the study agents reduced the frequency of Tregs overall, the authors noted an increase in CTLA4^+^ Tregs with drug exposure. The examination of the CD8^+^ T cell compartment noted a decreased frequency of TIM3^+^CD8^+^ T cells with exposure to belinostat, both alone and in combination, with the degree of diminishment associating with the response to therapy. The frequency of PD1^+^CD8^+^ T cells was also noted to decline with treatment, though the changes in this subset were not associated with the response or survival.

Subsequent preclinical and translational studies sought to explore the implications of epigenetic alteration on TET disease characteristics and identify novel avenues for therapy. Alterations in non-coding RNA were evaluated by Ganci et al., who performed miRNA expression profiling; they identified 87 miRNAs that were differentially expressed in thymic tumor tissue compared with normal controls and correlated with the histologic subtype [76]. miRNA profiles have also been noted to correlate with the disease characteristics of thymoma, particularly with the presence of thymoma-associated myasthenia gravis (TAMG). A study by Wang et al. noted decreased thymic stromal lymphopoietin (TSLP) mRNA expression [77]. Further investigation of the regulatory mechanisms governing aberrant expression of TSLP found the increased presence of miR-19b-5p in all thymoma patients compared with healthy controls, with a significant difference in levels between TAMG patients and thymoma patients without myasthenia gravis. The implications of these findings on the tumor immune microenvironment have yet to be explored, though the influence of TSLP on Th17 and interleukin (IL)-17 production has been reported. Additionally, imbalances of Th17 and Treg and ICOS^+^CD4^+^ T cell frequencies in the peripheral blood have been reported in TAMG patients [78,79].

Overall, these studies highlight the broad impacts of epigenetic modifications in thymic malignancies, suggesting that combinatorial strategies with epigenetic modulators may prove to impact both the tumor and mobilize the immune microenvironment to promote anti-tumor activity. The ideal combination, whether paired with other small molecule therapies, chemotherapies, or immune checkpoint blockade, needs to be supported with robust preclinical and translational studies that delineate the biologic mechanisms and establish biomarkers of response to improve patient selection for clinical trials.

## 5. Anti-Angiogenesis

Angiogenesis is a crucial component for maintaining the TME and promoting tumor growth and dissemination. The role of angiogenesis in thymic tumorigenesis was noted in a study by Tomita et al., who evaluated 46 surgically resected TETs via IHC and reported that the mean micro-vessel density correlated with tumor invasiveness, noting a higher expression of vascular endothelial growth factor (VEGF) in more aggressive TET histologies, such as invasive thymoma and thymic carcinoma, compared with non-invasive thymoma [80]. Additional studies have reported similar findings correlating more biologically aggressive TETs with greater micro-vessel density and platelet derived growth factor (PDGF)/VEGF/vascular endothelial growth factor receptor (VEGFR) expression [81,82,83,84,85]. Case reports highlighted the potential efficacy of oral anti-angiogenic TKIs in the TET patient population, including a thymic carcinoma patient harboring a *c-Kit* missense mutation who responded to sorafenib and several patients who underwent receptor tyrosine kinase analysis that revealed activation in PDGFβ and VEGF3 and subsequently received sunitinib and experienced partial responses in three out of four patients with metastatic thymic carcinoma that was refractory to standard therapies [86,87].

The results of these translational and case studies served as the catalyst for several trials that explored anti-angiogenesis agents, both alone and as combination therapies. Thomas et al. evaluated sunitinib, which is an oral multi-kinase inhibitor, including activity against VEGFR, KIT, and PDGF, in a phase 2 study of patients with advanced thymic tumors who progressed after at least one previous platinum-containing chemotherapy regimen [21]. In this study, 16 patients with thymoma and 24 patients with thymic carcinoma received 50 mg sunitinib daily for four weeks of a 6-week cycle. They reported ORRs of 6% and 26% in the thymoma and thymic carcinoma arms, respectively. The DCR was 91% in the thymoma patients and 81% in the thymic carcinoma patients. Given the poor activity seen in the thymoma arm, the cohort was closed to enrollment for the remainder of the study, per the protocol. A total of 70% of patients experienced grade 3 and 4 treatment-related adverse events, with the most common of these toxicities being lymphocytopenia, fatigue, stomatitis, and a reduction in left ventricular ejection fraction. A total of 13% of patients in the thymoma cohort developed autoimmune disorders while on treatment: one with pure red cell aplasia and one with hypogammaglobulinemia.

Correlative studies for this trial included gene sequencing of tumor tissue; immune profiling of the peripheral blood; and analysis of circulating endothelial cells, endothelial progenitor cells, and circulating tumor cells. Mutations in *TP53* and *DNMT3A* were the most common alterations of the 19 somatic variations affecting the 12 genes noted on the molecular profiling of 22 patients with available tissue. A significant increase in CTLA4^+^CD8^+^ T cells was noted in the total study population. Fifteen patients who experienced an increase of CTLA4^+^CD8^+^ T cells above the median were noted to have better overall survival compared to the remaining patients below the median. A total of 19 out of 28 patients were noted to have a non-significant increase in PD1 expression amongst Tregs, which remained relatively unchanged across the first three cycles of treatment. The study noted that the four patients who experienced a decrease in the PD1-expressing Tregs on day 1 of cycle 3 had significantly worse overall survival compared with the other 18 patients.

Further evaluation of the anti-angiogenesis drugs was reported in two phase 2 studies: lenvatinib in patients with advanced or metastatic thymic carcinoma (REMORA) and regorafenib in patients with B2—B2 thymoma and thymic carcinoma (Resound) [22,23]. REMORA consisted of eight institutions in Japan and enrolled thymic carcinoma patients who had received at least one platinum-based chemotherapy regimen. Forty-two patients received lenvatinib, which is a multi-kinase inhibitor (VEGFR1–3, FGFR1–4, PDGFRα, RET, and c-Kit), at 24 mg once daily in a 4-week cycle with the potential for several dose reductions in the event of unacceptable toxicities. The study reported an ORR of 38% with a DCR of 95%. The study authors noted a median PFS of 9.3 months and a 12-month OS of 83%. As expected, given the mechanism of action, common toxicities included hypertension, thrombocytopenia, diarrhea, proteinuria, and palmar–plantar erythrodysesthesia syndrome.

The Resound trial evaluated regorafenib, which is a VEGFR1–3, PDGFR-β, and FGFR inhibitor, in patients with B2—B3 thymoma and thymic carcinoma patients who had received prior standard-of-care platinum-based chemotherapy and administered 160 mg daily for three weeks of a 4-week cycle. The study reported responses via the Response Evaluation Criteria in Solid Tumors (RECIST), which utilizes anatomic measurement of tumor size, and the Choi Criteria, which includes both the size of the tumor and the presence of drug-induced necrosis [88]. Several studies employed the Choi criteria in the evaluation of angiogenic inhibitors, given the potential of these drugs to enhance tumor necrosis while the tumor size is unchanged [89,90]. The results of the Resound study showed a large variation between the two disease evaluation methods, with an ORR of 10% for thymoma and 0% for thymic carcinoma with RECIST versus 70% for thymoma and 86% for thymic carcinoma with Choi. The differences in ORR results were largely attributed to the shift of patients deemed to have stable disease by RECIST to the partial responses by Choi. The median PFSs were 9.6 months and 9.2 months for thymoma and thymic carcinoma, respectively; these were somewhat similar to the results of the REMORA study, which only included thymic carcinoma. Additionally, the toxicity profile was largely reflective of the drug class and included stomatitis, palmar–plantar erythrodysesthesia syndrome, nausea/vomiting, and diarrhea, though the frequency of hypertension was less compared with other agents.

The impact of anti-angiogenesis on the cytotoxic effects of conventional chemotherapy and immune cell trafficking has been reported across numerous preclinical and translational studies [91,92,93]. Combination anti-VEGF therapies with conventional chemotherapy have been employed in colorectal, lung, and gastric malignancies [94,95,96,97]. Wang et al. reported on a retrospective analysis of previously untreated thymoma and thymic carcinoma who received gemcitabine and cisplatin (GC) or GC with Endostar, which is an anti-angiogenic agent, between 2008 to 2017 [98]. They noted an ORR of 75% for patients who received GC with Endostar compared with 42.9% for patients who received GC alone, though no statistically significant differences were noted in the median PFS and OS between the two treatment groups. The lack of difference was attributed to the relatively small sample sizes. The RELEVENT trial is an ongoing phase 2 study evaluating the addition of ramucirumab, which is an anti-VEGFR2 agent, in combination with carboplatin and paclitaxel in the front-line treatment of locally advanced or metastatic thymic carcinoma or B3 thymoma with areas of carcinoma [99].

Preclinical models have suggested a synergy between anti-angiogenic agents and immunotherapy. Impaired lymphocyte trafficking via dysfunctional immune cell adhesion and arrested development of antigen-presenting dendritic cells have been implicated in tumor-promoting microenvironments [100]. The CAVEATT trial was a single-arm, phase 2 study that evaluated the combination of axitinib, which is a second-generation multi-kinase inhibitor with selective activity against VEGFR1–3, with avelumab, which is a PD-L1 inhibitor [101]. Patients with advanced WHO subtype B3 thymoma and thymic carcinoma who had progressed after at least one line of platinum-based chemotherapy were eligible, including patients who had previously received anti-angiogenesis agents. A total of 32 patients were enrolled in the study (27 thymic carcinoma, 5 B3 thymoma/combined histology), with 13 (41%) having received a prior anti-angiogenic treatment (e.g., sunitinib, ramucirumab, regorafenib). The study reported an ORR of 34%, with all the patients in this category experiencing a partial response (11 patients) and a DCR of 91%. The median OS was 26.6 months, with post hoc analysis revealing a median OS of 26.6 months amongst anti-angiogenic naïve patients versus 17.0 months in patients previously treated with anti-angiogenesis agents. Despite the combination of these two agents, the safety profile was generally manageable, with hypertension being the most common grade 3 adverse event (19%). Immune related adverse events (IRAEs) were seen in four patients (12%) and included a case of grade 3 interstitial pneumonitis and three cases of polymyositis (two grade 3 and one grade 4). All IRAEs were treated with immunosuppressive agents, though two cases required hospitalization. Correlative studies revealed no association between PD-L1 tissue expression and outcomes (e.g., ORR and PFS). Targeted exome sequencing re-demonstrated genomic alterations seen in previous studies, with alterations in *TP53* (33%) and *BAP1* (19%) being the most common. *PBMR1*, *TP53*, and *PI3KCA* mutations appeared to associate with the response, though the small sample size limited the extrapolation of these genes as predictive biomarkers.

Despite the scientific rationale for combination therapy, single-agent anti-angiogenic trials have shown modest response rates with an emphasis on disease control. The toxicity profile of these agents must also be weighed against the potential benefit. For thymoma patients, the indolent natural history associated with many of the subtypes makes the use of these agents particularly difficult. However, for thymic carcinoma, the results of the single-agent studies offer a potential opportunity for meaningful improvements. Several ongoing studies will add to our growing understanding of these agents, combined with an immune checkpoint blockade. The PECATI trial (NCT04710628) is a phase 2 study evaluating pembrolizumab in combination with lenvatinib in patients with WHO B3 thymoma and thymic carcinoma [25]. A study of sunitinib and pembrolizumab (NCT03463460) is ongoing in the United States, with a similar patient population [26]. Vorolanib, which is a VEGFR/PDGFR inhibitor, with nivolumab (NCT03583086) is also under investigation in a phase 1/2 study [27,102].

## 6. IGFR-PI3K-AKT-mTOR Pathway

The Insulin-Like Growth Factor 1 Receptor (IGF-1R) signaling pathway is a tightly regulated network critical for cell proliferation and has been implicated as an oncogenic driver in breast cancer, sarcoma, and NSCLC [103]. The activation of IGF-1R results in downstream signaling across numerous pathways, including phosphatidyl-inositol-3 kinase (PI3K)-AKT—mammalian target of rapamycin (mTOR) and mitogen activated protein kinase (MAPK). IGF-1R expression via IHC analyses has been reported across several studies, though the implication of IGR-1R presence and outcomes remains uncertain [104,105]. The clinical investigation of monoclonal antibodies targeting IGF-1R in early-phase studies was also reported during this time, with two case reports highlighting some evidence of therapeutic efficacy [106,107].

Rajan et al. conducted a phase 2 study of cixutumumab, which is an IGF-1R monoclonal antibody, in patients with advanced thymomas and thymic carcinomas who had previously received platinum-containing chemotherapy and reported an ORR of 14% in 37 patients with thymoma, with a DCR of 89% [29]. There were no responses seen in the thymic carcinoma cohort among the 12 patients accrued, resulting in cohort closure during the study. The median PFS and OS were 9.9 months and 27.5 months, respectively, within the thymoma cohort. Common adverse events associated with cixutumumab included hyperglycemia, hyperuricemia, anorexia, fatigue, and muscle cramping. Grade 3 elevations in serum lipase were noted in three patients (6%), all of whom required dose reductions. Of note, nine patients (24%) developed new-onset autoimmune disorders or worsening of a pre-existing autoimmune disorder while on the study drug. Induction of peripheral free IGF-1 was seen after administration of one dose of cixutumumab, with no association noted between the IGF-1 concentrations and response to therapy nor the development or exacerbation of autoimmune disease. Serum antibody analysis of autoimmune disease patients revealed significantly higher concentrations of anti-interferon (IFN)γ antibodies compared with baseline and a decreased frequency of IL-17-expressing CD4^+^ T cells. The study reported that the use of cixutumumab downregulated IGF-1R in surrogate PBMCs, which was both statistically significant and sustained, though no detectable post-treatment changes were seen in phospho-AKT expression. Responders were noted to have a significant increase in IFNγ-expressing CD4^+^ T cells, decreases in circulating endothelial progenitor cells, and increases while on treatment and higher titers of anti-granulocyte-macrophage colony-stimulating factor antibodies at baseline.

Molecular analysis of TETs demonstrated aberrations and the activation of downstream pathways, such as PI3K-AKT-mTOR, which may serve as an explanation for the limited efficacy of IGF-1R inhibition in the previous study [6,108,109,110]. Preclinical analyses of pictilisib, which is a pan-PI3K inhibitor, and rapamycin, which is a mTOR inhibitor, in TET cell lines reduced epithelial cell proliferation and viability [109,110]. Zucali et al. reported on a phase 2 study of 10 mg everolimus daily in patients with advanced thymoma and thymic carcinoma after first-line treatment [30]. They reported ORRs of 9% and 15% and DCRs of 94% and 78% for thymoma and thymic carcinoma, respectively. The median PFSs were 16 and 5.6 months for thymoma and thymic carcinoma, respectively. Frequent adverse events included stomatitis (66%), fatigue (48%), mucositis (36%), and pneumonitis (36%). Grade 3 and 4 toxicities were noted in 28% of patients and commonly included liver toxicity, neutropenia, and metabolic disorders. A total of 70% of patients required dose interruption and 28% required dose reduction. Three deaths were reported due to pneumonitis (two infectious, one non-infectious).

Prior molecular testing noted elevated phospho-AKT activity and the downregulation of PTEN in WHO type A/AB in C19MC-positive thymomas compared with normal tissues [108]. Based on this data and the modest efficacy seen with everolimus, a single-arm phase 2 study evaluated buparlisib, which is a pan-PI3K inhibitor, in TET patients with relapsed or refractory disease [31]. Fourteen thymoma patients (WHO type B2/3) received 100 mg buparlisib daily. The study found an ORR of 7.1% with a DCR of 50%. The median PFS and OS were 11.1 and 40.0 months, respectively. Fatigue, anorexia, pruritus, acneiform rash, anxiety, cough, and nausea were the common adverse events. Grade 3 and 4 adverse events included dyspnea, rash, and liver toxicity. Molecular analysis of the C19MC cluster and exome-sequencing did not reveal overexpression of this cluster, nor did it reveal significant alterations predictive of a PI3K inhibition response.

## 7. Cell Cycle Regulation/STAT 3 Pathway

Chromosomal alterations associated with aberrant cell cycle regulation have been reported in TETs and are associated with specific histological subtypes, with thymic carcinomas harboring more chromosomal gains and losses and the involvement of larger foci [111,112]. Alterations resulting in the loss of heterozygosity on 6q25.2–25.3 and 6p21.31 (major histocompatibility locus) were found to be the most frequent chromosomal aberration [113,114]. Loss of the 6p23 region resulted in the copy number loss of *FOXC1*, which is a known tumor growth suppressor gene, and is associated with a shorter time to progression [115]. Deletions and rearrangements in chromosome 6 have been reported in several human malignancies, including breast cancer and melanoma [116,117,118,119]. DNA methylation alterations in TETs, including at the promoter region of *CDKN2A/B* and subsequent inactivation of p16INK4A and RB, may play a role in tumorigenesis and poor prognosis [120,121,122].

The tropomyosin receptor kinase (Trk) family of transmembrane receptors, which consists of TrkA (*NTRK1*), TrkB (*NTRK2*), and Trk3 (*NTRK3*), regulates nervous system development and the maintenance of neuronal tissue. Somatic oncogenic fusions have been associated with approximately 1% of all solid tumors and commonly arise from *NTRK1* and *NTRK3* genes [123]. Neurotropin receptor expression was evaluated in 99 surgically resected TET patients (88 with thymoma, 11 with thymic carcinoma) via IHC analysis [124]. The authors reported the near-universal expression of cytoplasmic TrkA, while no tumors expressed TrkB or TrkC. Additionally, the study noted the expression of neutrotrophin receptor p75^NTR^ across nearly all tumors, with the pattern of expression correlating with histological subtypes of thymoma. Ozono et al. reported the expression of Trk receptors in 48 surgically resected TET tissue samples (43 thymomas and 5 thymic carcinomas), noting 10% and 14% of cases expressing TrkA and TrkB, respectively [125]. All five cases of thymic carcinoma exhibited high TrkB expression and an associated high expression with more advanced disease based on the Masaoka–Koga stage. A phase 1 trial evaluated milciclib (PHA-848125AC), which is a pan inhibitor of the cyclin D-dependent kinases Src and TrkA, in patients with advanced or metastatic solid tumors and noted a partial response and stable disease in two patients with thymic carcinoma, resulting in further evaluation [126].

Two phase 2 multicenter trials evaluated milciclib, CKDO-125A-006 and CKDO-125A-007 [32]. The 006 trial evaluated milciclib in 72 TET patients (20 with B3 thymoma and 52 with thymic carcinoma) who had received one prior line of treatment. This study reported an ORR of 4.2% and a DCR of 72.2%.The 007 trial evaluated milciclib in 30 TET patients (17 with B3 thymoma and 13 with thymic carcinoma) who had received multiple lines of chemotherapy. Similarly, this study also reported an ORR of 3.3% and a DCR of 70.0%. The median PFS and OS for 006 trial were 5.78 and 24.44 months, respectively, while 007 trial reported 5.65 and 21.03 months, respectively. An ongoing phase 1 study (NCT03556228) is evaluating the selective TrkA inhibitor, namely, VMD-928, in TrkA-overexpressed solid tumors, including thymic carcinoma [35].

An additional study tested the role of palbociclib, which is a CDK4/6 inhibitor, in the treatment of relapsed or refractory TETs [34]. Forty-eight patients received 125 mg palbociclib daily for 21 days of a 28-day cycle. Though the study predominantly investigated B3 thymoma (27%) and thymic carcinoma (48%), it also included A-B2 thymomas (23%), with the remaining histologies being unknown. The ORR was 12.5%, with four thymoma patients, and two thymic carcinoma patients achieving partial responses. The DCR was 79.2%, with approximately half of the patients who achieved stable disease remaining on treatment for longer than 12 months. The median PFS and OS were 11.0 and 26.4 months with no significant differences observed between the thymoma and thymic carcinoma cohorts. The most common toxicities associated with treatment were neutropenia, anemia and thrombocytopenia, fever, fatigue, anorexia, and diarrhea. The most common grade 3 and 4 adverse events included hematologic (54.2%) and pneumonitis (4.2%). Dose reductions were required for 14 patients (29.2%) and were primarily due to adverse events of neutropenia, anemia, and pneumonitis. Exploratory biomarker analysis was conducted for 12 patients (5 with thymoma, 7 with thymic carcinoma) with available tumor tissue. Six cell cycle or DNA damage repair genes (*WEE1*, *POLE2*, *TGFB2*, *IL1R1*, *FANCC*, and *MYD88*) were associated with durable clinical benefit to palbociclib.

Clinical studies have also attempted to explore the efficacy and safety of selinexor, which is a selective inhibitor of nuclear export, based on the results of human cell line testing, which showed Exportin 1 (XPO1) expression in two thymoma cell lines (IU-TAB1 and T1682) and three thymic carcinoma cell lines (Ty82, MP57, and T1889) [127]. The use of selinexor, which is an XPO1 inhibitor, showed impaired TET cell proliferation and survival, and the induction of p53-dependent and independent cytotoxic effects. The study also uncovered potential resistance mechanisms, including the loss of p53 activity and XPO1 gene amplification. IHC analysis of 132 TET tumors and 16 normal thymic tissues revealed an association between a higher expression of XPO1 and more aggressive histologic subtypes (e.g., B2/B3 thymoma and thymic carcinoma) and advanced stages of disease. Data from a phase 1 study of selinexor was notable for a patient with thymoma who achieved a partial response and remained on treatment for almost 2 years [128]. As a result of this data, two phase 2 studies were initiated to evaluate the role of selinexor in the treatment of TETs: the SELECT trial based in the United States and the TET-SEL trial based in Europe. Unfortunately, both studies, which were planned to evaluate selinexor in TET patients who progressed after treatment with at least one platinum-based chemotherapy regimen, were prematurely closed.

Signal transducer and activator of transcription (STAT) proteins play crucial roles in mediating cellular signaling for cell proliferation and differentiation [129]. Hyperactivation of STAT3 on tumor cells has been associated with immunosuppressive microenvironments. IHC studies demonstrated the presence of overactivated STAT3 pathways in thymic malignancies, especially in more aggressive histologic subtypes of thymoma and thymic carcinoma [130,131]. A phase 1b study of napabucasin, which is an orally administered reactive oxygen species generator that modulates the STAT3 pathway, in combination with paclitaxel was conducted in a heavily pre-treated TET population with advanced, unresectable, or metastatic disease [33]. The results of 16 patients (10 with thymoma, 6 with thymic carcinoma) were reported with an ORR of 67% and a DCR of 83% for thymoma and an ORR of 25% and a DCR of 63% for thymic carcinoma. Grade 3 adverse events included diarrhea, vomiting, and abdominal pain. The development of STAT3 inhibitors in the field of oncology as a whole has largely been abandoned, though the complete results of this study may spur further investigation.

## 8. Conclusions

The landscape of targeted therapies for thymic tumors is characterized by a variety of responses across histologic subtypes and significant toxicities for many of the most effective agents. Promising agents with manageable toxicities often fell short due to a lack of activating mutations or bypass pathways conferring resistance. Combinatorial strategies are attempting to augment the modest efficacy of two proven agents while evaluating the safety profile. The development of safety biomarkers will be necessary for moving these combinations into widespread use. Molecularly directed strategies will continue to evolve as our drug development and delivery systems advance.

## Figures and Tables

**Table 1:** Select previous and *ongoing* clinical trials that have evaluated molecularly directed therapies in advanced TETs. SSTR—somatostatin receptor; *—per RECIST criteria; EGFR—epidermal growth factor receptor; IGFR—insulin-like growth factor receptor; PRRT—peptide receptor radionuclide therapy; PAC—cisplatin, doxorubicin, and cyclophosphamide; CT—carboplatin and paclitaxel; T—thymoma; TC—thymic carcinoma; NET—neuroendocrine tumor; UNK—unknown; ORR—overall response rate; DCR—disease control rate; PFS –progression-free survival; OS—overall survival; NR—not reached.

Reference/Study	Treatment	N	ORR (%)	DCR (%)	Median PFS (Months)	Median OS (Months)
SSTR Targeting Agents Palmieri et al. (2002) [10] Loehrer et al. (2004) [11] *NCT05918302* [12] *NCT04375267* [13]	Octreotide + Prednisone Octreotide + Prednisone *Everolimus* + *PRRT* *Everolimus* *Olaparib + PRRT*	16 (10 T, 3 TC, 3 NET) 38 (32 T, 5 TC, 1 NET) *120* (*solid tumor*) *18* (*solid tumor*)	38 32 (38 T, 0 TC/NET)	75 68	14 9.2	15 NR
EGFR Targeting Agents Kurup et al. (2005) [14] Bedano et al. (2008) [15] *NCT01025089* [16]	Gefitinib Erlotinib + Bevacizumab *Cetuximab + PAC*	26 (19 T, 7 TC) 18 (11 T, 7 TC) *18 (T, TC)*	4 0	58 60	- -	- -
c-KIT Targeting Agents Salter et al. (2008) [17] Giaccone et al. (2009) [18]	Imatinib Imatinib	11 (TC) 7 (2 T, 5 TC)	0 0 (100 T, 0 TC)	27 29	- 2	- 4
Epigenetic Targeting Agents Giaccone et al. (2011) [19] Thomas et al. (2014) [20]	Belinostat Belinostat + PAC	41 (25 T, 16 TC) 25 (11 T, 14 TC)	5 (8 T, 0 TC) 40 (64 T, 21 TC)	68 (79 T, 50 TC) 100 (93 T, 96 TC)	5.8 (11.4 T, 2.7 TC) 9.0 (NR T, 7.2 TC)	19.2 (NR T, 12.4 TC) 28.5 (NR T, 21.4 TC)
Anti-Angiogenic Agents Thomas et al. (2015) [21] Sato et al. (2020) [22] Perrino et al. (2022) * [23] Conforti et al. (2022) [24] *NCT04710628* [25] *NCT03463460* [26] *NCT03583086* [27] NCT03694002 [28]	Sunitinib Lenvatinib Regorafenib Axitinib + Avelumab *Lenvatinib* + *Pembrolizumab* *Sunitinib* + *Pembrolizumab* *Vorolanib* + *Nivolumab* *CT* + *Ramucirumab*	41 (16 T, 25 TC) 42 (TC) 19 (11 T, 8 TC) 32 (5 T, 27 TC) *43* (*T*, *TC*) *30* (*TC*) *88* (*solid tumor*) *66*	6 T, 26 TC 38 37 (10 T, 0 TC) 34 (40 T, 33 TC)	81 T, 91 TC 95 79 (90 T, 86 TC) 91 (100 T, 89 TC)	8.5 T, 7.2 TC 9.3 9.6 (9.6 T, 9.2 TC) 7.5	15.5 T, NR TC NR 33.8 (NR T, 20.1 TC) 26.6
IGFR-PI3K-AKT Agents Rajan et al. (2014) [29] Zucali et al. (2018) [30] Abu Zaid et al. (2022) [31]	Cixutumumab Everolimus Buparlisib	49 (37 T, 12 TC) 50 (32 T, 18 TC) 14 (T)	10 (14 T, 0 TC) 12 (9 T, 17 TC) 7	78 (89 T, 42 TC) 88 (94 T, 78 TC) 50	8.2 (9.9 T, 1.7 TC) 10.1 (16.6 T, 5.6 TC) 11.1	16.2 (27.5 T, 8.4 TC) 25.7 (NR T, 14.7 TC) 40.0
Cell Cycle/STAT3 Agents Besse et al. (2018) [32] Besse et al. (2018) [32] Kalra et al. (2018) [33] Jung et al. (2023) [34] *NCT03556228* [35]	Milciclib Milciclib Napabucasin Palbociclib *VMD-928*	72 (20 T, 52 TC) 30 (17 T, 13 TC) 16 (10 T, 6 TC) 48 (24 T, 23 TC, 1 UNK) *74 (solid tumor or lymphoma)*	4.2 3.3 25 T, 67 TC 13 (17 T, 9 TC)	72 70 63 T, 83 TC 79 (79 T, 78 TC)	5.8 5.7 - 11.0 (13 T, 9.2 TC)	24.4 21.0 - 26.4 (26.4 T, 25.6 TC)
